# Ground beetle fauna (Coleoptera, Carabidae) of Mordovia State Nature Reserve (Russia)

**DOI:** 10.3897/BDJ.9.e69807

**Published:** 2021-10-25

**Authors:** Alexander Ruchin, Sergey Alekseev, Leonid Egorov, Oleg Artaev, Gennadiy Semishin, Mikhail Esin

**Affiliations:** 1 Joint Directorate of the Mordovia State Nature Reserve and National Park "Smolny", Saransk, Russia Joint Directorate of the Mordovia State Nature Reserve and National Park "Smolny" Saransk Russia; 2 Ecological club "Stenus", Kaluga, Russia Ecological club "Stenus" Kaluga Russia; 3 Prisursky State Nature Reserve, Cheboksary, Russia Prisursky State Nature Reserve Cheboksary Russia; 4 Papanin Institute for Biology of Inland Waters Russian Academy of Sciences, Borok, Russia Papanin Institute for Biology of Inland Waters Russian Academy of Sciences Borok Russia

**Keywords:** biodiversity, Coleoptera, Carabidae, pitfall traps, Republic of Mordovia

## Abstract

**Background:**

Protected areas are organised in different climatic zones, which usually include typical ecosystems characteristic of certain climatic zones. In most cases, protected areas are biodiversity hotspots. These areas are benchmarks in terms of nature conservation and to determine their biological diversity is becoming an important task. It is important to investigate the carabid family of protected areas within the framework of understanding the overall biological diversity of these systems. In addition, ground beetles, as one of the largest groups of ground-based inhabitants, are indicators of the state of ecosystems and serve as markers of their well-being.

**New information:**

We present 2,969 new occurrence records comprising 226 species of Carabidae, belonging to eight subfamilies, from the Mordovia State Nature Reserve (central Russia). Ten species are listed for the first time for the Mordovia State Nature Reserve fauna after previous research: *Cicindelamaritima*, *Bembidionstriatum*, *Dyschiriusangustatus*, *Dyschiriusarenosus*, *Notiophilusaestuans*, *Bembidionargenteolum*, *Bembidionvelox*, *Bradycelluscaucasicus*, *Cymindisangularis* and *Syntomustruncatellus*, five of which were first recorded for the Republic of Mordovia (Egorov et al. 2020). Previously, this information was not published anywhere and we wanted to make it available to everyone by embedding it in the global database on biodiversity (GBIF).

## Introduction

Anthropogenic disturbances, such as urbanisation, toxic chemical pollution, more frequent and intense fires, deforestation and climate change have recently had a significant impact on biodiversity in many ecosystems. Insects play an important role in the functioning of ecosystems and a decrease in their biodiversity can cause serious disruptions of natural processes. However, there is a lack of data on the diversity, abundance, phenology and other biological traits of many insect groups that play a crucial role in ecosystems (Butchart et al. 2010, Bondarenko et al. 2020). Ground beetles (Coleoptera, Carabidae) are important predators of other insects and are considered economically useful in agricultural systems (Sklodowski and Garbalinska 2011, Ruchin et al. 2019, Rozhnov et al. 2019). As ground beetles are diverse and abundant in the ground layer of landscapes with varying degrees of disturbance, they have been used as bioindicators to assess ecosystem health (Rainio and Niemelä 2003, Noordijk et al. 2008, Venn et al. 2013, Zamotajlov et al. 2019). Thus, documenting ground beetle diversity and distribution in natural ecosystems, such as the Mordovia State Nature Reserve, provides critical baseline data that can be used for future comparisons with more disturbed sites. Additionally, these data can be used in bioindication, for a general understanding of ecosystem biodiversity and for the protection of individual species and ecosystems.

From the published dataset, five taxa are recommended for inclusion in the Red Data Book of the region (*Carabusnitens*, *Calosomainquisitor*, *Carabusaurolimbatus*, *Carabusschoenherri* and *Lebiamarginata*). These are rare and protected species. At the same time, *Lebiamarginata* was found within the region only within the territory of the Mordovian Reserve.

## Sampling methods

### Study extent

The Mordovia State Nature Reserve is located in the Republic of Mordovia (Central Russia). Its area is 321.62 km^2^. Forests cover 89.3% of the Mordovia State Nature Reserve. The main type of forest is pine (*Pinussylvestris*) forests. Pure and mixed stands of pine dominate the southern, central and western parts. Birch (*Betulapendula*) forests grow in burned areas and are the second most common forest type. Deciduous forests with *Quercusrobur* and *Tiliacordata* are located in small areas in the northern, western and south-western parts. Small patches of forest, dominated by *Piceaabies* and *Alnusglutinosa*, are located mainly in the floodplains of small rivers and streams (Khapugin et al. 2016, Ruchin and Makarkin 2017).

### Quality control

Each observation includes fundamental information, such as location (latitude/longitude), date, name of observer and name of the identifier. Coordinates were determined on site with a GPS device (various models of smartphones) or after the fact using Google Maps. We used conventional methods for collecting ground beetles, including hand-collecting, pitfall traps, light trapping and partial beer traps (Golub et al. 2012, Ruchin et al. 2020). Pitfall traps (the primary method) were deployed during April-August (sometimes September) 2008-2020. The traps were 0.5-litre cups containing 200 ml of 4% formalin solution. In each habitat, we installed 10 traps along a transect with 2-3 m between consecutive traps. The collected material was determined by S. Alekseev and L. Egorov. Determination was carried out according to Isaev (2002), MÜLLER-MOTZFELD (2004). We followed the nomenclature proposed in Kryzhanovskij et al. (1995),Lobl and Lobl (2017).

## Geographic coverage

### Description

The dataset provides new records of Carabidae (Coleoptera) from the territory of the Mordovia State Nature Reserve (Russian Federation).

### Coordinates

54°42'24"N and 54°56'08"N Latitude; 43°04'28"E and 43°37'49"E Longitude.

## Taxonomic coverage

### Description

All individuals of Carabidae were identified to the species level. The taxonomic diversity of the study area is represented by 226 species belonging to eight subfamilies. Ten species are listed for the first time for the Mordovia State Nature Reserve fauna in this study. According to the literature (Ruchin et al. 2019, Egorov et al. 2020), another 15 species are known in the research area and not captured during our studies. Given the long-term nature of our focused research, this is an almost exhaustive list of species that have breeding populations in Mordovia State Nature Reserve (241 species) (Alekseev et al. 2021).

### Taxa included

**Table:** 

Rank	Scientific Name	
phylum	Arthropoda	
class	Insecta	
order	Coleoptera	
family	Carabidae	
subfamily	Carabinae	
subfamily	Elaphrinae	
subfamily	Harpalinae	
subfamily	Loricerinae	
subfamily	Nebriinae	
subfamily	Patrobinae	
subfamily	Scaritinae	
subfamily	Trechinae	

## Temporal coverage

**Data range:** 2008-4-01 – 2020-6-07.

## Usage licence

### Usage licence

Other

### IP rights notes

Creative Commons Attribution (CC-BY) 4.0 License

## Data resources

### Data package title

Carabidae of Mordovia State Nature Reserve

### Resource link

https://www.gbif.org/dataset/4b901e9a-18c4-473c-a56d-b7689b55ecbc

### Alternative identifiers

https://doi.org/10.15468/9s9z7s

### Number of data sets

1

### Data set 1.

#### Data set name

Carabidae of Mordovia State Nature Reserve

#### Number of columns

21

#### 

**Data set 1.** 

Column label	Column description
occurrenceID	The globally unique identifier number for the record
basisOfRecord	The specific nature of the data record: HumanObservation
eventDate	date format as YYYY-MM-DD
scientificName	The full scientific name including the genus name and the lowest level of taxonomic rank with the authority
kingdom	The full scientific name of the kingdom in which the taxon is classified
phylum	The full scientific name of the phylum or division in which the taxon is classified
class	The full scientific name of the class in which the taxon is classified
order	The full scientific name of the order in which the taxon is classified
family	The full scientific name of the family in which the taxon is classified
decimalLatitude	The geographic latitude of location in decimal degrees
decimalLongitude	The geographic longitude of location in decimal degrees
coordinateUncertaintyInMetres	The horizontal distance (in metres) from the given decimalLatitude and decimalLongitude describing the smallest circle containing the whole of the Location
geodeticDatum	The ellipsoid, geodetic datum or spatial reference system (SRS) upon which the geographic coordinates given in decimalLatitude and decimalLongitude are based
Country	The name of the country or major administrative unit in which the Location occurs
countryCode	The standard code for the country in which the Location occurs
individualCount	The number of individuals present at the time of the Occurrence
year	Year the event was recorded
month	The month the event was recorded
day	The integer day of the month on which the Event occurred
recordedBy	A person or group responsible for recording the original Occurrence
identifiedBy	A list of names of people, who assigned the Taxon to the subject
